# The disease burden and health-related quality of life in Chinese children with genetic cholestatic liver diseases: a cross-sectional study of progressive familial intrahepatic cholestasis and Alagille syndrome

**DOI:** 10.1186/s13023-026-04365-8

**Published:** 2026-04-27

**Authors:** Yuji Jiang, Weiyuan Fang, Mingzi Cen, Jihu Li, Jianshe Wang, Jinxi Ding, Ye Chen

**Affiliations:** 1 School of International Pharmaceutical Business, China Pharmaceutical University, Nanjing, China; 2 Pediatric Liver Center, Children’s Hospital of Fudan University, Shanghai, China; 3 Pharmaceutical Market Access Policy Research Center, School of International Pharmaceutical Business, China Pharmaceutical University, Nanjing, China

**Keywords:** Genetic cholestatic liver diseases, Rare diseases, Pediatric cholestasis, Burden of disease, Influencing factor

## Abstract

**Background:**

Genetic cholestatic liver diseases (GCLDs), including progressive familial intrahepatic cholestasis (PFIC) and Alagille syndrome (ALGS), are rare inherited liver disorders that manifest in early childhood and lead to substantial morbidity and healthcare utilization. However, comprehensive data on their economic and humanistic burden in China are scarce.

**Methods:**

A cross-sectional study was conducted in 2024 among caregivers of GCLD children recruited from a major specialized referral center in China. Data were collected *via* an online questionnaire covering demographic and clinical profiles, diagnostic pathways, healthcare resource use, direct and indirect costs, and health-related quality of life (HRQoL) using the PedsQL™ Inventory. Caregiver burden was assessed using a Numerical Rating Scale. Univariate and multiple linear regression analyses were performed to identify factors associated with economic burden and HRQoL.

**Results:**

Among the 170 patients (PFIC: 41.2%, ALGS: 58.8%), misdiagnosis occurred in 62.4% of cases. The mean age at first visit was 3.9 months, while the mean age at confirmed diagnosis was 11.2 months. The average annual total cost was CNY 244,700 per patient, with direct medical, direct non-medical, and indirect costs accounting for 60.6%, 11.1%, and 28.3% respectively. Both univariate and multivariate analyses revealed that older age and higher family income were associated with better PedsQL scores, whereas disease severity, complications and more frequent follow-up significantly predicted poorer HRQoL.

**Conclusions:**

GCLDs confer a significant economic and quality-of-life burden on affected families in China, compounded by diagnostic delays and high out-of-pocket expenses. These findings underscore the need for improved diagnostic accuracy, early intervention, more coordinated rare disease care networks and enhanced psychosocial and financial support policies for this vulnerable population.

## Introduction

Intrahepatic cholestasis (IHC) refers to a spectrum of conditions characterized by impaired bile flow within the liver, which can result from diverse etiologies including infections, pregnancy, medications, metabolic disorders and genetic defects [1]. Among these, inherited forms of IHC, caused by pathogenic mutations affecting bile synthesis, transport or regulation, represent a distinct subset of rare and severe cholestatic liver diseases that often present in infancy or childhood [2, 3]. These genetic disorders are particularly challenging due to their early onset, progressive nature, and limited treatment options, leading to substantial morbidity and mortality [4, 5]. Recently, this concept has been formalized in the 2024 Clinical Practice Guidelines of the European Association for the Study of the Liver (EASL), which uses the term Genetic Cholestatic Liver Diseases (GCLDs) to classify these disorders and provides updated diagnostic and therapeutic frameworks [6]. Internationally, major GCLD subtypes commonly outlined in guidelines or consensuses include progressive familial intrahepatic cholestasis (PFIC), Alagille syndrome (ALGS), benign recurrent intrahepatic cholestasis (BRIC), cystic fibrosis-associated liver disease (CFLD) and other inborn errors of bile acid synthesis [6–9].

Mirroring these international diagnostic advancements, the clinical management and recognition of GCLDs in China have undergone a rapid evolution, primarily driven by the progressive establishment of national rare disease policies. A critical milestone was the inclusion of ALGS in the first National Rare Disease List in 2018, followed by the addition of PFIC to the second list in 2023 [10, 11]. This systemic recognition has effectively bridged the gap between global clinical standards and domestic practice, significantly enhancing physician awareness and diagnostic standardization across the country. Consequently, these policy developments have not only improved diagnostic accuracy through greater access to genetic testing but also facilitated the preliminary development of targeted reimbursement pathways and improved orphan drug accessibility.

Among these recognized disorders, PFIC caused by mutations in genes such as *ATP8B1*, *ABCB11*, *MYO5B* and *ABCB4*, has an estimated incidence of one in 50,000 to one in 100,000 live births [12, 13], making up around 10% to 15% of pediatric cholestatic cases [14]. While ALGS, primarily associated with variants in *JAG1* or *NOTCH2*, occurs in approximately one in 30,000 to one in 70,000 births [15, 16]. These two disorders were selected as the focus of this study due to their high local prevalence, well-defined genetic basis, characteristic clinical profiles, significant disease burden and heightened recognition within China’s national health policy framework.

Clinically, GCLDs often manifest in infancy with jaundice, intense pruritus, hepatosplenomegaly and darkened urine. Without effective intervention, the disease may progress to cirrhosis and end-stage liver disease, necessitating liver transplantation [17, 18]. Patients typically present with severe pruritus and persistent hyperbilirubinemia, while ALGS is additionally characterized by distinctive facial features, congenital heart defects and vertebral abnormalities [19, 20]. Long-term follow-up studies indicate development of progressive liver damage and increased mortality compared with the general pediatric population [5, 21].

Current management of pediatric intrahepatic cholestasis primarily relies on pharmacological and surgical interventions. Ursodeoxycholic acid is typically used as the first-line pharmacologic therapy, which demonstrates efficacy in improving key biochemical parameters in majority of patients [9, 22, 23]. For patients unresponsive to medical therapy, surgical options such as partial external biliary diversion and liver transplantation can effectively reduce bile acid accumulation and improve clinical symptoms. However, liver transplantation can be associated with substantial risks, including surgical complications, lifelong immunosuppression, and considerable financial burden [24, 25]. Recently, ileal bile acid transporter (IBAT) inhibitors such as odevixibat and maralixibat have emerged as promising targeted therapies, demonstrating significant reductions in serum bile acid levels and pruritus in international clinical trials [26, 27]. However, the annual cost of such treatment could exceed patient’s affordability and create profound financial barriers for most families.

The burden imposed by GCLD extends beyond clinical manifestations to encompass substantial economic and quality-of-life challenges. Pruritus, one of the most debilitating symptoms, leads to sleep disturbances, skin excoriations, and significantly impaired daily functioning [21, 28]. Caregivers of children with GCLD also report considerable mental and physical strain, disruptions in personal and professional lives, and reduced overall well-being [29]. Economically, the heavy costs associated with chronic management, frequent hospitalizations, and loss of income could contribute to a high incidence of catastrophic health expenditures. Studies have indicated that families of children with rare diseases bear substantial multi-dimensional economic burdens which extend beyond direct healthcare costs to encompass indirect productivity losses and out-of-pocket expenditures [30]. In China, disparities in healthcare access and genetic testing availability exacerbate these burdens, especially in rural areas, often leading to financial hardship. Despite these challenges, there is a notable lack of large-scale, systematic studies in China evaluating the multidimensional burden of GCLDs, with existing literature predominantly focused on clinical and genetic aspects rather than patient-centered or economic outcomes. This research gap not only hinders evidence-based policymaking but also potentially masks systemic health inequities in the allocation of specialized resources, a challenge documented in other developing healthcare contexts where rare or complex conditions often face inappropriate hospital resource distribution [31].

To address this critical evidence gap, we conducted a cross-sectional study aiming to characterize the clinical and diagnostic profiles of GCLD patients in China, estimate the economic burden and incidence of catastrophic health expenditure, and assess health-related quality of life in affected children and their caregivers. Our findings intend to inform health policies and contribute to the development of a comprehensive care framework for patients with GCLDs in China.

## Methods

### Study design and enrolment criteria

A cross-sectional survey was conducted to evaluate the disease burden and health-related quality of life (HRQoL) among children with GCLDs in China. Given the heterogeneous classification of GCLDs (or IHCs) across different countries, we selected PFIC and ALGS as representative models for GCLDs due to their inclusion in China’s National Rare Disease Directories with well-characterized genetic backgrounds, thereby reducing diagnostic uncertainty and improving the validity of our burden estimates.

Subjects were confirmed as ALGS by standard clinical criteria and the presence of a disease-causing mutation in *JAGGED1* or *NOTCH2*. Eligible patients fulfil histopathological evidence of paucity or absence of interlobular bile ducts, accompanied by at least three characteristic clinical features, including chronic cholestasis, cardiac murmur, butterfly vertebrae, ocular abnormalities, renal abnormalities, or typical facial features. Patients with PFIC were defined by biochemical evidence of cholestasis for greater than six months, with the presence of any relevant mutant alleles of *ATP8B1*, *ABCB11*, *ABCB4*, *MYO5B*, or *TJP2*, without another definable cause of cholestasis. Patients with co-occurring rare diseases were excluded to minimize confounding factors that could skew the disease-specific cost and HRQoL data, thereby ensuring that the reported burden is uniquely representative of the GCLD population. Other exclusion criteria encompassed all other non-genetic cholestatic disorders, severe comorbidities (e.g., malignancies), and incomplete data entries.

### Patient recruitment and data collection

A primary investigation on patient registration was conducted among three relevant national rare disease organizations: the China Rare Disease Alliance, Illness Challenge Foundation (ICF), and Alagille Angels Care Home. These organizations consistently identified the Children’s Hospital of Fudan University (CHFU) as the predominant institution providing specialized care and long-term follow-up for the GCLD patient population in China. As a National Children’s Medical Center, it manages a substantial and geographically diverse cohort of GCLD patients referred from across China, providing a representative analytic sample for this national investigation. Based on this finding, our study targeted this center and consecutively recruited caregivers of children with clinically confirmed GCLDs who attended its outpatient clinics from January to December 2024. The sample size was determined a priori to ensure sufficient power for the multivariable linear regression analysis. Based on Green’s rule of thumb and Cohen’s power analysis criteria, minimum sample size of 118 was deemed necessary to achieve a statistical power of 0.80 for estimated 10 predictors with a medium effect size. The structured electronic questionnaire underwent a three-stage validation process before being released. First, it was reviewed for content validity by three senior pediatric hepatologists through in-depth interviews. Subsequently, a pilot study involving the research team, clinical staff and patient families was conducted to ensure the clarity of medical and financial questions. Technical trials and feedback channel were also tested to ensure smooth release of the survey. Trained researchers and attending physicians jointly administered the finalized questionnaires during clinic visits. Given the young age of participants, all responses were proxy-reported by primary caregivers. Electronic informed consent was obtained prior to survey initiation. The study protocol was approved by the Ethics Committee of Children’s hospital of Fudan University (Approval No. APHFU-2020402).

### Questionnaire

The structured questionnaire was designed to comprehensively capture clinical, socioeconomic, and humanistic outcomes. It consisted of three main sections, beginning with a combined module collecting basic demographic characteristics alongside detailed diagnostic and treatment history. This included information such as patient age, sex, residence, household income, insurance coverage, age at initial diagnosis, presenting symptoms, clinical complications, and records of pharmacological or surgical interventions. Disease severity was classified into three tiers based on clinical presentation, aligning with the Guidelines for the Management of Cholestatic Liver Diseases (2021) issued by the Chinese Society of Hepatology [9]. Severe cases were defined by the presence of prolonged jaundice (> 6 months) accompanied by frequent pruritus, as the chronic persistence of these symptoms is a recognized indicator of significant disease burden and progressive liver injury in this guideline. Moderate cases involved persistent jaundice with only intermittent or mild pruritus, while the mild tier comprised the remaining patients who did not meet these thresholds. This symptom-based stratification was employed to capture the long-term clinical phenotype, prioritizing the symptomatic drivers that directly determine health-related quality of life and daily family functioning.

The second section provided a detailed assessment of economic burden. Direct medical costs encompassed outpatient, inpatient, pharmaceutical, and diagnostic expenditures. Direct non-medical costs included transportation, accommodation, and nutrition-related expenses. Indirect costs captured productivity loss and opportunity costs incurred by caregivers. Catastrophic health expenditure was defined according to World Health Organization criteria as out-of-pocket health payments exceeding 40% of a household’s annual income [32].

The final section evaluated health-related quality of life using two validated instruments. The Pediatric Quality of Life Inventory 4.0 Generic Core Scales (PedsQL) [33], a 23-item proxy-reported tool, measured physical, emotional, social, and school functioning on a zero to 100 scale. Caregiver burden was assessed using the Numerical Rating Scale (NRS), through which respondents rated anxiety, depression, and fatigue over the preceding month on a scale from zero to 10, where higher scores reflect greater psychological strain. The overall health-related quality of life was summarized using mean and standard deviation values derived from both the PedsQL and the NRS scores, where higher scores on each instrument reflect better health outcomes.

### Data analysis

The questionnaires were submitted *via* the cloud-based platform *Wenjuanxing* following a comprehensive proofreading process. Upon completion of data collection, the raw dataset was exported and assessed for logical consistency, and subsequent data cleaning was conducted using Excel. Data cleaning included a systematic identification of outliers using Tukey’s method to prevent undue influence on the model estimates. The distributions of both direct costs and PedsQL scores were evaluated using the Shapiro-Wilk test, complemented by visual inspection of histograms. The annual cost-related outcomes exhibited a right-skewed distribution. To more accurately represent central tendency and mitigate the influence of outliers, the median and interquartile range (IQR) were selected as the primary measures for cost data.

Between-group differences in health outcomes were analyzed using either Analysis of Variance (ANOVA) or the Kruskal-Wallis test, depending on whether parametric assumptions were met. To examine associations between PedsQL scores (a continuous measure bounded between zero and 100) and potential predictors, a generalized linear model was employed, which accommodates skewed distributions and restricted-range outcomes [34]. Predictors included both non-clinical factors such as age, sex, residence, insurance status, income, and incidence of catastrophic health expenditure, as well as clinical variables including history of misdiagnosis, disease severity, presence of complications, and hospitalization frequency. Among which, the age cutoff of 4 years was selected based on the natural history of GCLDs, as children beyond this age are more likely to experience advanced complications or become candidates for surgical intervention [16, 21]. Variables that reached statistical significance (*P* < 0.05) in univariate analyses were included in a multivariable generalized linear model to identify independent correlates of PedsQL scores while adjusting for potential confounding factors. Prior to multivariable modeling, multicollinearity among independent variables was assessed using the Variance Inflation Factor (VIF). All included predictors exhibited VIF values between 1.0 and 1.5, indicating no significant multicollinearity. All analyses were performed using SPSS version 27.

## Results

### Basic information of participants

The study cohort included 170 pediatric patients diagnosed with GCLDs, consisting of 70 cases of PFIC and 100 cases of ALGS. Among them, 98 (57.65%) were male and 72 (42.35%) were female, with a median age of 3.88 years. Age distribution analysis indicated that the majority of participants were under four years of age and of preschool status, consistent with the early-onset nature of GCLDs. The ages of enrolled patients ranged from three months to 18 years. Geographically, a slight majority of patients (51.76%) resided in rural areas. Participants represented a national cross-section, with 48.82% residing in East China, 25.88% in Central China, and 25.29% in West China.

Insurance coverage analysis revealed near-universal enrolment in basic medical insurance (94.71%, *n* = 161). In contrast, supplementary insurance coverage was limited: only 28.82% (*n* = 49) possessed commercial insurance. Additionally, 5.88% (*n* = 10) were covered by critical illness insurance programs and 3.53% (*n* = 6) were enrolled in social assistance schemes—both supported by regional government initiatives. Household income distribution reflected notable socioeconomic diversity. The majority of families (78.82%, *n* = 134) reported an annual income of less than 100,000 CNY, while 14.12% (*n* = 24) earned between 100,000 and 200,000 CNY, and 7.06% (*n* = 12) had incomes exceeding 200,000 CNY per year (Table 1).

**Table 1:** Demographics and characteristics of patients with GCLDs (*N* = 170)

Variables	*N*	Ratio
**Sex**		
Male	98	57.65%
Female	72	42.35%
**Disease type**		
PFIC	70	41.18%
ALGS	100	58.82%
**Age**		
≤ 4	86	50.59%
> 4	84	49.41%
**Residence**		
Urban	82	48.24%
Rural	88	51.76%
**Region**		
East China	83	48.82%
Central China	44	25.88%
West China	43	25.29%
**Education status**		
Pre-school	115	67.65%
Enrolled in school	55	32.35%
**National insurance coverage**		
Yes	161	94.71%
No	9	5.29%
**Additional insurance program (non-exclusive)**		
Commercial insurance	49	28.82%
Critical illness insurance	10	5.88%
Social assistance supporting rare diseases	6	3.53%
None of the above	115	67.65%
**Annual family income (ten thousand CNY)**		
≤ 10	134	78.82%
10–20	24	14.12%
> 20	12	7.06%

**Fig. 1:**
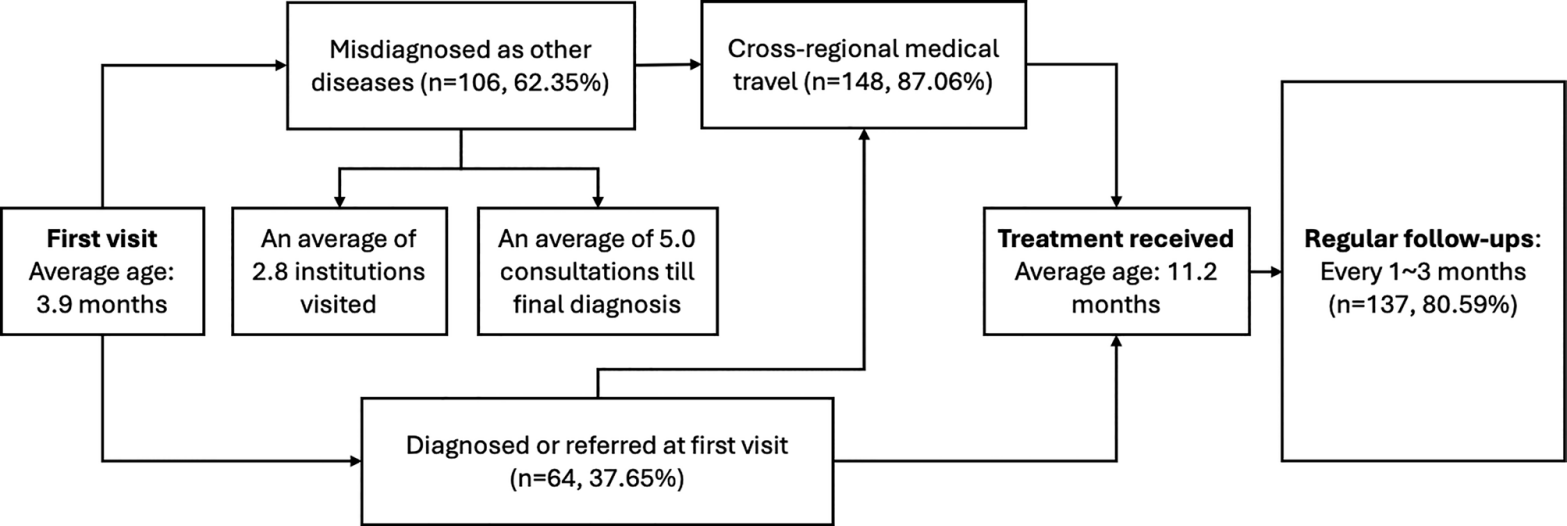
Diagnostic journey and treatment pattern of GCLD patients

### Diagnosis and treatment

**Fig. 2:**
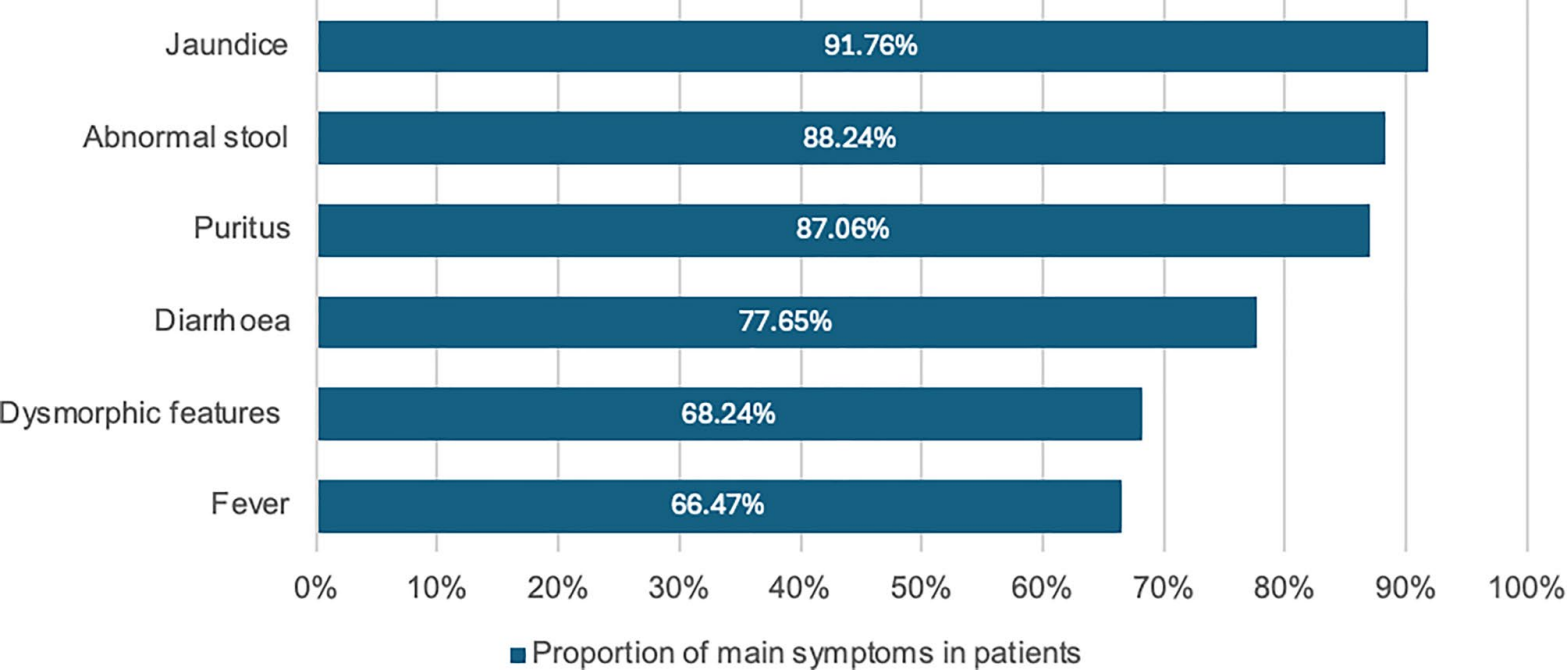
Main symptoms of GCLD patients

The diagnostic evaluation of 170 pediatric patients with GCLDs was visualized in Fig. 1 and revealed significant challenges in disease recognition and management. Among the cohort, 106 patients (62.35%) were initially misdiagnosed, while only 64 (37.65%) received a correct diagnosis or appropriate referral during their first medical consultation. The median age at symptom onset was 3.9 months. The most common clinical manifestations included jaundice (*n* = 156, 91.76%), abnormal stool characteristics (*n* = 150, 88.24%), and pruritus (*n* = 148, 87.06%), as shown in Fig. 2. Patients visited a mean of 2.8 different healthcare institutions and underwent approximately five clinical visits before obtaining a definitive diagnosis. The most prolonged diagnostic pathway involved four institutions and 19 separate visits.

Substantial diagnostic delays were observed, with a median time to diagnosis of 7.3 months and a maximum delay of 5.5 years. Initial clinical assessments most frequently occurred in departments of hepatology or infectious diseases (*n* = 84, 49.41%), general pediatrics (*n* = 38, 22.35%), and gastroenterology (*n* = 22, 12.94%). The most frequent misdiagnoses included cytomegalovirus or toxoplasmosis infection (*n* = 33, 19.41%), inborn errors of metabolism (*n* = 32, 18.82%), and biliary atresia (*n* = 20, 11.76%).

Regarding treatment patterns, conventional pharmacotherapy was universally administered, with ursodeoxycholic acid used in 166 patients (97.65%), cholestyramine in 138 (81.18%), and rifampicin in 59 (34.71%). Additional therapeutic agents included fibrates (*n* = 60, 35.29%), glycyrrhizin (*n* = 48, 28.24%), traditional Chinese medicine (*n* = 36, 21.18%), and IBAT inhibitors (*n* = 17, 10.00%). Surgical interventions, including partial external biliary diversion and liver transplantation were performed in 23 cases (13.53%).

The median age at treatment initiation was 11.2 months. Majority of patients (80.59%) maintained regular follow-up visits at 1–3 month intervals for ongoing monitoring. Notably, 87.06% of families reported traveling across regions to access specialized care for their child’s rare disease, reflecting disparities in medical resource distribution. Cross-regional medical access was identified as a barrier to continuity of care by 39.39% of non-adherent patients (*n* = 13). Furthermore, financial burden was also cited as a major obstacle to consistent follow-up by 27.27% of these families (*n* = 9).

### Economic burden

The economic burden analysis revealed a substantial financial impact on families of children with GCLDs, as shown in Table 2. The total annualized economic burden per patient averaged 24.47 ten thousand CNY, with a median of 11.40 ten thousand CNY (IQR: 5.97,17.59). Direct medical costs constituted the largest proportion of total expenses, accounting for 60.6% of the burden, with a median value of 4.42 ten thousand CNY (IQR: 3.22, 7.40). Outpatient services represented the most direct medical expenditures, comprising 83.0% of this category, while hospitalization costs accounted for the remaining 17.0%. A further breakdown of these expenditures reveals that specialized medications including choleretic drugs and supportive therapies constituted the largest outpatient component of 2.91 ten thousand CNY (92.2%), followed by laboratory examinations and diagnostic tests (5.4%) and regular follow-up fees (2.3%). Hospitalization costs were analyzed for 115 patients (67.7%) with ≥ 1 admission in the past year. The median annual expenditure in this subgroup was 1.50 ten thousand CNY (0.6, 3.0).

Direct non-medical costs, including transportation, accommodation, and nutrition-related expenses, represented 11.1% of the total economic burden, with a median expenditure of 1.56 ten thousand CNY (0.59, 3.50). Indirect costs, primarily resulting from productivity loss due to caregiving responsibilities and work absenteeism among parents, also represented a significant portion of the total burden at 28.3%, with a median value of 3.90 ten thousand CNY (0.05, 6.61).

Financial catastrophe was nearly universal among study participants when applying the WHO catastrophic health expenditure (CHE) thresholds, as reflected in Table 3. Using income-stratified capacity-to-pay calculations based on China’s 2024 economic statistics, we found 86.47% of families (147/170) exceeded the 40% expenditure-to-income ratio defining CHE. The burden displayed clear socioeconomic gradients, with low-income households (annual income < 50,000 CNY) experiencing 98.61% CHE incidence versus 41.67% in high-income groups (> 200,000 CNY). This disparity persisted despite 94.71% coverage by basic medical insurance, revealing critical gaps in financial protection for rare disease management.

Families employed multiple coping strategies to manage these costs. While 55.29% (94/170) relied solely on household income, 38.24% (65/170) resorted to borrowing, accumulating median debts of 100,000 CNY (IQR: 50,000, 150,000). 7.06% (12/170) utilized charitable platforms, though these provided limited relief with a median amount of 15,000 CNY. Another 11 families raised a median of 26,000 CNY from crowdfunding projects. The economic impact extended beyond direct medical expenses, as 82.94% of caregivers (141/170) reported income reduction due to caregiving responsibilities, including 58.82% (100/170) who quit employment entirely and 80.59% (137/170) taking extended unpaid leave.

**Table 2:** Annualized direct and indirect costs of GCLD management (10 thousand CNY)

Categories	Mean	%	Median	IQR
Direct medical cost	14.83	60.6%	4.42	(3.22, 7.40)
Outpatient cost	12.32	83.0%	3.26	(3.03, 4.51)
Hospitalization cost	2.51	17.0%	1.00	(0, 2.40)
Direct non-medical cost	2.71	11.1%	1.56	(0.59, 3.50)
Indirect cost	6.93	28.3%	3.90	(0.05, 6.61)
Total economic burden of disease	24.47	100.0%	11.40	(5.97, 17.59)

**Table 3:** Catastrophic health expenditure (CHE) by income group

Income quintile	Household capacity-to-pay* (CNY)	CHE threshold (CNY)	Families with CHE *n*/*N* (%)
Low (< 50,000 CNY)	17,550	7,020	71/72 (98.61)
Lower-middle (50,000–79,999 CNY)	39,742	15,897	32/37 (86.49)
Middle (80,000-129,999 CNY)	62,396	24,958	27/32 (84.38)
Upper-middle (130,000-199,999 CNY)	98,140	39,256	12/17 (70.59)
High (≥ 200,000 CNY)	181,733	72,693	5/12 (41.67)
Total	-	-	147/170 (86.47)

*Household capacity-to-pay = Annual income − basic living expenses (food/housing) per China 2024 Statistical Yearbook

### Health-related quality of life

The assessment of quality of life (QoL) among children with GCLDs and their caregivers revealed considerable health-related burdens across the cohort as shown in Table 4. Caregivers reported moderate levels of burden as measured by NRS, with mean scores of 5.74 (± 3.03) for depression, 7.11 (± 2.68) for anxiety, and 7.35 (± 2.64) for fatigue. Meanwhile, the mean overall PedsQL Generic Core Scale score for the patients was 73.65 (± 18.94), indicating a moderate impairment in health-related quality of life. When stratified by annual disease-related direct cost, significant variations in QoL were observed (*P* < 0.05). Children from families with annual costs below 50,000 CNY had a notably higher overall PedsQL score (84.46 ± 17.14), whereas those with costs between ¥100,000–200,000 scored significantly lower (68.67 ± 19.24), suggesting that higher financial burden is associated with poorer patient QoL.

**Table 4:** Quality-of-life measurement for GCLD children (*n* = 165)

Annual cost (10k CNY)	*N* (%)	Caregiver burden in NRS scale (mean ± SD)			Patients’ PedsQL score (mean ± SD)			
		Depression	Anxiety	Fatigue		Physiology	Psychology	Overall
< 5	33 (20.0%)	4.3 ± 3.16	5.73 ± 3.04	5.85 ± 3.02		81.98 ± 17.98	86.09 ± 19.35	84.46 ± 17.14
5–10	29 (17.6%)	5.72 ± 2.83	6.97 ± 2.81	7.17 ± 2.8		72.03 ± 19.32	81.81 ± 13.85	77.72 ± 15.02
10–20	75 (45.5%)	6.41 ± 2.81	7.77 ± 2.2	8.2 ± 1.95		67.04 ± 20.28	69.65 ± 20.72	68.67 ± 19.24
> 20	28 (17.0%)	5.64 ± 3.19	7.11 ± 2.78	7 ± 2.83		68.88 ± 22.07	70.55 ± 19.57	70.04 ± 18.41
Total	165 (100.0%)	5.74 ± 3.03	7.11 ± 2.68	7.35 ± 2.64		71.22 ± 20.61	75.23 ± 20.3	73.65 ± 18.94

Abbreviations: NRS, Numerical Rating Scale; PedsQL, Pediatric Quality of Life Inventory

The univariate analysis of variance (ANOVA) was conducted to identify factors associated with health-related quality of life, as measured by the PedsQL Generic Core Scales among the 165 children with GCLDs (Table 5). Several demographic and socioeconomic variables demonstrated significant associations with PedsQL scores. Notably, age was a strong influencing factor (*F* = 48.819, *P* < 0.001), with children older than four years exhibiting markedly higher PedsQL scores (82.41 ± 15.61) compared with those aged four years or younger (64.57 ± 17.84). Place of residence also showed a significant effect (*F* = 4.368, *P* = 0.038), as children from urban areas reported a better quality of life (76.76 ± 17.45) than those from rural areas (70.66 ± 19.92). Furthermore, annual family income was significantly associated with PedsQL outcomes (*F* = 6.550, *P* = 0.002). Children from families with an annual income exceeding 200,000 CNY had the highest score (89.58 ± 10.10), followed by those in the 100,000–200,000 CNY group (78.83 ± 16.85), while those with incomes of 100,000 CNY or less had the lowest scores (71.27 ± 19.10). Conversely, sex, national insurance coverage, and participation in additional insurance programs did not show statistically significant effects on quality of life (*P* > 0.05).

**Table 5:** Results of analysis of variance of sociodemographic factors for health utility scores among GCLD children (*n* = 165)

Variables	*N*	Mean ± SD	F	*P*
**Sex**			1.930	0.167
Male	95	71.90 ± 19.82		
Female	70	76.03 ± 17.53		
**Age**			48.819	<0.001*
≤ 4	81	64.57 ± 17.84		
> 4	84	82.41 ± 15.61		
**Residence**			4.368	0.038*
Urban	81	76.76 ± 17.45		
Rural	84	70.66 ± 19.92		
**National insurance coverage**			0.298	0.586
Yes	156	73.46 ± 19.14		
No	9	77.01 ± 15.56		
**Whether involved in an additional insurance programme**			1.308	0.254
Yes	53	76.10 ± 18.88		
No	112	72.49 ± 18.94		
**Annual family income**			6.550	0.002*
≤ 10	130	71.27 ± 19.10		
10–20	23	78.83 ± 16.85		
>20	12	89.58 ± 10.10		
**Annual disease-related direct cost (10k CNY)**			6.733	<0.001*
≤ 5	33	84.46 ± 17.13		
5–10	29	77.72 ± 15.02		
10–20	75	68.67 ± 19.24		
>20	28	70.04 ± 18.41		
**Whether annual expense exceed CHE threshold**			11.644	<0.001*
Yes	142	71.69 ± 18.53		
No	23	85.77 ± 17.21		

Abbreviations: CHE, catastrophic health expenditure

*P<0.05

Clinical characteristics and disease-related factors were also significantly associated with PedsQL scores as presented in Table 6. Disease type influenced quality of life (*F* = 4.751, *P* = 0.031), with patients diagnosed with ALGS reporting higher scores (76.28 ± 18.68) than those with PFIC (69.81 ± 19.80). Genetic subtype was another significant factor (*F* = 2.991, *P* = 0.013). Apart from the two patients which exhibited relatively high scores with *TJP2* mutations, patients with *ABCB4* mutations had the highest PedsQL scores (83.14 ± 20.49), whereas those with *ATP8B1* mutations had the lowest (59.14 ± 16.13). Disease severity significantly impacted quality of life (*F* = 3.868, *P* = 0.023), with mildly affected children scoring highest (83.16 ± 16.47), followed by those with moderate (72.36 ± 18.09) and severe disease (71.64 ± 19.61). The presence of complications was a strong negative predictor (*F* = 13.318, *P* < 0.001), as patients without complications reported significantly better PedsQL scores (78.28 ± 16.67) than those with complications (67.82 ± 20.10). Treatment duration was also significantly associated with outcomes (*F* = 12.385, *P* < 0.001), where children treated for more than five years had the highest scores (83.03 ± 14.76), and those with two years or less of treatment had the lowest (67.43 ± 17.73). Additionally, a higher number of annual hospitalizations (≥ 3 times) was linked to lower PedsQL scores (65.46 ± 17.82) compared with fewer hospitalizations (< 3 times, 75.78 ± 18.70; *F* = 8.373, *P* = 0.004). Similarly, higher follow-up frequency was associated with better quality of life (*F* = 6.550, *P* = 0.002). In contrast, a history of misdiagnosis, delay in diagnosis, and whether the patient had undergone surgery did not demonstrate statistically significant effects on PedsQL scores (*P* > 0.05)

**Table 6:** Results of analysis of variance of diagnostic and clinical factors for health utility scores among GCLD children (*n* = 165)

Variables	*N*	Mean ± SD	F	*P*
**Type**			4.751	0.031*
ALGS	98	76.28 ± 18.68		
PFIC	67	69.81 ± 19.80		
**Genetic subtype**			2.991	0.013*
* NOTCH2/JAG1*	98	76.28 ± 18.68		
* ABCB11*	21	73.38 ± 13.03		
* ATP8B1*	16	59.14 ± 16.13		
* MYO5B*	10	68.13 ± 23.93		
* ABCB4*	7	83.14 ± 20.49		
Other/Unspecified	13	71.29 ± 20.31		
**Experience of misdiagnosis**			1.387	0.241
Yes	101	72.27 ± 19.04		
No	64	75.83 ± 18.72		
**Gap from symptom occurrence to final diagnosis**			1.350	0.247
Within 1 year	141	74.36 ± 18.63		
Longer than 1 year	24	69.51 ± 20.57		
**Severity**			3.868	0.023*
Mild	25	83.16 ± 16.47		
Moderate	61	72.36 ± 18.09		
Severe	79	71.64 ± 19.61		
**Presence of complications**			13.318	<0.001*
Yes	73	67.82 ± 20.10		
No	92	78.28 ± 16.67		
**Treatment duration**			12.385	<0.001*
≤ 2 years	69	67.43 ± 17.73		
2–5 years	45	71.06 ± 21.13		
> 5 years	56	83.03 ± 14.76		
**Number of annual hospitalization stay(s)**			8.373	0.004*
< 3 times	131	75.78 ± 18.70		
≥ 3 times	34	65.46 ± 17.82		
**Follow-up frequency**			6.550	0.002*
≤ 10	130	71.27 ± 19.10		
10–20	23	78.83 ± 16.85		
>20	12	89.58 ± 10.10		
**Received surgery or not**			1.296	0.257
Yes	20	69.14 ± 19.06		
No	145	74.28 ± 18.90		

*P < 0.05

To further delineate the independent predictors of QoL, a multiple linear regression model was constructed (Table 7). The analysis identified five significant factors influencing PedsQL scores. Older age group (*β* = 0.383, *P* < 0.001) and higher family income (*β* = 0.162, *P* = 0.012) were associated with improved QoL. In contrast, greater disease severity (*β*=−0.158, *P* = 0.013), higher follow-up frequency which indicates more severe or unstable disease (*β*=−0.178, *P* = 0.018), and the presence of complications (*β*=−0.267, *P* < 0.001) were negatively associated with QoL. The model explained a substantial proportion of variance in QoL outcomes, underscoring the multifaceted impact of socio-demographic and disease-specific factors on the well-being of children with GCLDs

**Table 7:** Multiple linear regression results of factors influencing patients’ quality of life

Independent variable	Non-standard conversion coefficient		Standard coefficient	T	*P*
	Regression coefficient	Standard error			
(Constant)	62.892	7.291		8.626	< 0.001
X1 Age group	14.453	2.747	0.383	5.261	< 0.001
X2 Family income	5.166	2.037	0.162	2.536	0.012
X3 Severity	−4.125	1.643	−0.158	−2.511	0.013
X4 Follow-up frequency	−3.936	1.639	−0.178	−2.401	0.018
X5 Presence of complications	−10.136	2.383	−0.267	−4.253	< 0.001

## Discussion

This study presents a comprehensive analysis of the socioeconomic and clinical determinants influencing the health-related quality of life and economic burden experienced by pediatric patients with GCLDs in China. The results demonstrate substantial disparities in disease burden attributable to demographic, economic, and clinical factors, highlighting an urgent need for integrated health policy and clinical management strategies

The demographic characteristics of the cohort marked by early disease manifestation and significant regional variability in healthcare access have highlighted the pressing need for structured newborn screening programs and early diagnostic protocols. Early onset necessitates prompt intervention to alter disease progression and improve long-term outcomes. Geographic disparities in healthcare resources compound these challenges, particularly for rural families who must travel extensively for specialized care. The inadequate insurance coverage for critical illness and rare diseases further exacerbates these inequities, limiting access to high-cost treatments such as liver transplantation and innovative pharmacotherapies. These findings underscore the urgency of policy reforms aimed at expanding insurance schemes to include rare disease management, potentially modeled after successful frameworks in countries with advanced rare disease support systems [35].

The economic analysis indicates that GCLDs pose a considerable financial burden on affected families, as evidenced by a high incidence of CHE, reflecting opportunities for enhancing financial risk protection. The mean annual cost identified in this study (244,700 CNY, approximately 34,000 USD) represents a staggering economic challenge when viewed against China’s 2024 macroeconomic indicators. According to the 2024 Statistical Communiqué on National Economic and Social Development released by the National Bureau of Statistics, the GDP per capita was 95,749 CNY, and the national per capita disposable income was 41,314 CNY. Thus, the annual management cost of GCLD is equivalent to 2.6 times the GDP per capita and nearly 6 times the average individual’s annual disposable income. This disproportionate ratio confirms that GCLD-related expenses constitute a catastrophic health expenditure for most affected families, potentially leading to long-term financial instability or poverty.

Direct medical costs accounted for approximately 60% of the total economic burden, highlighting a significant structural shortcoming in current coverage. While the inclusion of PFIC and ALGS in the National Rare Disease Lists provide critical institutional recognition, this status remains largely nominal in terms of financial benefits, as it does not translate into automatic drug reimbursement. Financial protection for GCLD families is ultimately contingent upon a treatment’s inclusion in the National Reimbursement Drug List (NRDL). For GCLD patients, the CHE arises from the long-term, continuous need for outpatient drugs. The high out-of-pocket direct costs observed here underscore the current gap between policy recognition and financial subsidization, where reimbursement caps and a limited scope of covered medications fail to accommodate the high-cost orphan drugs necessary for GCLD management. Importantly, caregiver employment disruption, observed in 82.94% of families, amplifies this economic strain through lost productivity and reduced income mobility, highlighting the need to incorporate these often-overlooked indirect costs into future health policy assessments. To strengthen support for families affected by rare diseases, a multi-layered health security framework could be beneficial. Building on basic insurance, complementary financing pathways such as commercial health insurance and dedicated rare disease funds may help improve coverage for high-cost, low-prevalence conditions. Policy development may also focus on gradually expanding drug reimbursement catalogs and integrating tailored rare disease supports into broader health security planning, thereby better aligning with patient needs.

The evaluation of HRQoL elucidated key socioeconomic and clinical correlates. Given that socio-economic status remains a primary determinant of health inequalities [36], it is evident that economic fragility exacerbates the HRQoL burden for families managing GCLDs. The positive correlation between income levels, urban residence, and PedsQL scores indicates that economic stability is a prerequisite for effective burden mitigation, suggesting that financial protection policies are as essential as clinical interventions. This aligns with established findings in pediatric chronic disease research, where socioeconomic status often influences access to consistent, high-quality care and supportive resources [37]. Older age was also predictive of improved HRQoL, which may reflect a combination of developmental adaptation, more stable disease phases, or the cumulative benefit of long-term disease management. Conversely, disease severity, complications, and frequent hospitalizations were strongly associated with diminished quality of life. These results are consistent with studies of other chronic pediatric liver conditions, where clinical complexity significantly impairs physical and psychosocial functioning [38]. The particularly low scores among children with complications emphasize the importance of preventive care and early treatment to avoid disease-related deterioration. From a health economics perspective, it is established that investing in primary healthcare and preventive programs is significantly more cost-effective than addressing advanced complications [39]. Consequently, establishing standardized early-screening pathways and improving local clinical awareness for GCLDs could substantially reduce both clinical severity and the long-term economic burden on families.

From a health systems perspective, our findings highlight the importance of optimizing the allocation and accessibility of medical resources for rare diseases like GCLDs. Efforts to optimize healthcare infrastructure, including the effective allocation of resources, could help alleviate the financial strain on families and improve clinical outcomes. At present, the distribution of specialized medical resources remains uneven across regions, which disproportionately affects patients in rural or underserved areas. Nearly 90% of the families in our cohort reported having to travel across regions to access specialized diagnosis and treatment, reinforcing the need for more coordinated and tiered rare disease care networks. Notably, while our study benefits from capturing patients at a national referral hub, we acknowledge the inherent selection bias due to its single-center nature. Families who are unable to reach tertiary centers due to financial constraints or those whose children are misdiagnosed at local levels remain underrepresented. Patients belong to this category often face more severe economic and health challenges and this underscores the need for future studies integrated with primary healthcare data. The high rate of misdiagnosis suggests a critical gap in the early identification of GCLDs within the primary healthcare system. Many cases are initially mismanaged due to a lack of specialized knowledge among community pediatricians and limited regional access to molecular diagnostics. Drawing from the importance of primary healthcare measures in global health resilience [40], establishing a more robust screening and referral network at the primary care level is essential. Policy efforts could focus on strengthening regional collaboration, promoting the development of designated rare disease medical centers, and supporting the role of leading tertiary hospitals in providing technical guidance and training to improve early diagnosis and standardized treatment capabilities at the local level. Such measures would not only alleviate the financial and logistical burden on families but also enhance the overall efficiency and equity of rare disease healthcare delivery in China.

## Limitations

Several limitations should be considered when interpreting the results of this study. First, regarding the study design, the single-center recruitment at a national referral hub may introduce selection bias. Although our center serves patients nationwide, the sample might be skewed toward families with the financial resources to travel to Shanghai or those with more severe clinical phenotypes requiring specialized care, potentially underrepresenting rural or socioeconomically disadvantaged populations. Furthermore, as a cross-sectional study, it cannot establish causal relationships between the identified predictors and HRQoL outcomes. Longitudinal research is needed to track these dynamics over time. Second, in terms of data accuracy and methodology, our findings rely on caregiver-reported data, which introduces potential self-reporting and recall bias on financial spending. The use of proxy-reported PedsQL measures, while standard in pediatric research, may not fully capture the child’s subjective experience as perceived by the children themselves. Third, concerning the comparative scope, this study lacks a control group such as a healthy pediatric population. This absence limits our ability to benchmark the specific magnitude of the GCLD-induced burden against other health states. Fourth, regarding the clinical assessment, disease severity was categorized based on symptom frequency. The lack of objective biochemical parameters (e.g., serum bile acid levels) may introduce subjective bias. Finally, although the overall sample size is substantial for these rare diseases, it remains insufficient for robust conclusions within certain genetic subgroups (e.g., *n* = 2 for TJP2). This small subgroup size limits the power of our analysis, meaning that statistically significant findings in these categories should be interpreted with caution and viewed as exploratory. Despite these limitations, this study offers meaningful insights into the determinants of HRQoL in GCLD patients and provides a foundation for more targeted family-centered interventions. Future multicenter studies incorporating mixed-methods, objective health metrics, and control groups will be essential to further validate these findings.

## Conclusion

In conclusion, the management of GCLD requires a coordinated approach that combines clinical innovation with socio-economic support. Future efforts should focus on building evidence-based, patient-centered care pathways while advocating for policies that ensure equitable access to services and financial risk protection for all affected families. By enhancing financial protection and optimizing medical resource allocation for rare diseases such as GCLDs, China can move toward a more supportive and sustainable system that reduces familial hardship and improves long-term outcomes for pediatric patients.

In conclusion, this study characterizes the profound economic and humanistic burden of GCLDs in China. These findings establish a benchmark for families accessing specialized care, while suggesting even more severe hardships for underserved populations in remote areas who remain unable to reach high-tier centers. The management of GCLD thus requires a coordinated approach that combines clinical innovation with socio-economic support. Future efforts should focus on building evidence-based, patient-centered care pathways while advocating for policies that ensure equitable access and financial risk protection for all affected families. By optimizing medical resource allocation and enhancing the social safety net for rare diseases, China can move toward a more supportive system that reduces familial hardship and improves long-term outcomes for pediatric GCLD patients.

## Data Availability

Data are available upon reasonable request. All data relevant to the study are included in the article or uploaded as supplementary information.

## Abbreviations

ALGS: Alagille syndrome
ANOVA: Analysis of Variance
BRIC: Benign recurrent intrahepatic cholestasis
CFLD: Cystic fibrosis-associated liver disease
EASL: European Association for the Study of the Liver
GCLD: Genetic cholestatic liver disease
HRQoL: Health-related quality of life
IBAT: Ileal bile acid transporter
IHC: Intrahepatic cholestasis
NRDL: National Reimbursement Drug List
NRS: Numerical rating scale
PedsQL: Pediatric Quality of Life Inventory
PFIC: Progressive familial intrahepatic cholestasis
QoL: Quality of life
VIF: Variance Inflation Factor
